# Vibration-induced dynamic structural superlubricity on MoS_2_/Au(111) under ultrahigh vacuum

**DOI:** 10.3762/bjnano.17.71

**Published:** 2026-08-06

**Authors:** Shuyu Huang, Yiming Song, Antoine Hinaut, Gema Navarro-Marín, Antonio Cammarata, Ernst Meyer, Thilo Glatzel

**Affiliations:** 1 Department of Physics, University of Basel, 4056 Basel, Switzerland; 2 Department of Control Engineering, Faculty of Electrical Engineering, Czech Technical University in Prague, Technicka 2, 16627 Prague 6, Czech Republic

**Keywords:** atomic force microscopy (AFM), dynamic structural superlubricity, mechanical actuation, molybdenum disulfide, nanotribology

## Abstract

Achieving rapid and flexible control of friction across different material surfaces is of great significance and remains a longstanding challenging goal in modern tribology. Here, we demonstrate dynamic structural superlubricity in monolayer MoS_2_-coated gold surfaces induced by external mechanical actuation. By tuning the excitation frequency to the cantilever resonance, stick–slip motion is effectively suppressed, leading to a significant reduction in friction even under high-load conditions. Experimental results, supported by a modified phononic friction model, reveal that torsional vibrations reduce energy barriers and promote transitions between atomic sites. This strategy provides a universal and reversible approach for active friction control at the nanoscale.

## Introduction

Friction at the nanoscale governs the performance, reliability, and lifetime of a wide range of emerging technologies, including micro- and nanoelectromechanical systems (MEMS/NEMS), data storage devices, and atomically precise manufacturing [1]. Unlike macroscopic friction, which is often described by empirical laws, nanoscale friction arises from non-linear stick–slip dynamics and is strongly influenced by energy dissipation pathways [2], thermal fluctuations [3], and external actuation [4]. A central challenge in nanotribology, therefore, is not only to understand the origin of frictional forces at atomic interfaces but also to actively regulate them in a controllable and reversible manner. Over the past decades, a variety of strategies have been developed to manipulate frictional forces, ranging from mechanical regulation [5–7] and electrical control [8–9] to structural engineering [10–11], and thermal control [12].

Thermal approaches primarily act by modifying the energy landscape experienced by the sliding interface. Increasing temperature assists atoms in overcoming potential energy barriers, and friction is expected to vanish at infinitely slow relative speeds due to thermal activation [3,7]. It works only within specific low-speed regimes, and elevated temperatures can lead to local material softening or accelerated degradation and oxidation of the sliding interface. By leveraging structural engineering, specifically through moiré superlattice modulation and in-plane straining, it is possible to achieve ultralow friction by suppressing moiré-level deformation and optimizing contact quality [11,13–16]. However, this method strongly depends on the intrinsic structural parameters, which are often difficult to change dynamically once the device is fabricated. Recently, electrical control has emerged as an important route for tuning nanoscale friction, particularly in low-dimensional materials [17–18]. For instance, the use of electric field or currents enable to change the electronic states or electrostatic interactions at the interface, offering high flexibility and fast response times. Despite these promising results, electrically driven friction control presents several inherent limitations. It is highly dependent on the interfacial electronic properties [8]. Furthermore, the degree of friction reduction is often positively correlated with interfacial conductivity, making these methods much less flexible or effective for insulating systems. Regulation through electric current inevitably generates Joule heating. This leads to a local temperature rise at the interface, which can intensify energy dissipation and accelerate material oxidation and wear [19].

Alongside these approaches, mechanical excitation offers a distinct advantage as a universal method for friction regulation [20–22]. Unlike electrical approaches, mechanical actuation does not require conductive contacts and can be applied to a wide range of materials, including insulating systems [4,23]. Moreover, it not only provides high flexibility for dynamically controlling frictional dissipation at the interface but also avoids unwanted chemical reactions induced by heating or electric fields. Indeed, dynamic mechanical control has already demonstrated considerable success in bulk and three-dimensional systems, where oscillatory inputs can stabilize smooth sliding and reduce friction through energy landscape modulation [24–25]. However, extending these concepts to atomically thin materials present new challenges due to their reduced dimensionality, enhanced flexibility, and strong coupling between in-plane and out-of-plane degrees of freedom.

In this work, we focus on mechanical excitation with an aim to achieve active and reversible friction control in a single-layer two-dimensional material. By exploiting dynamic modulation of the interfacial energy landscape, we demonstrate the ability to switch between distinct frictional states, including a superlubric regime. This approach avoids limitations imposed by material properties and provides a platform to study fundamental friction mechanisms in low-dimensional systems. Our findings contribute to bridging the gap between established mechanical control strategies in bulk materials and the emerging field of friction engineering in atomically thin interfaces, offering new insights into the design of tunable and energy-efficient nanoscale devices.

## Results

**Figure 1:**
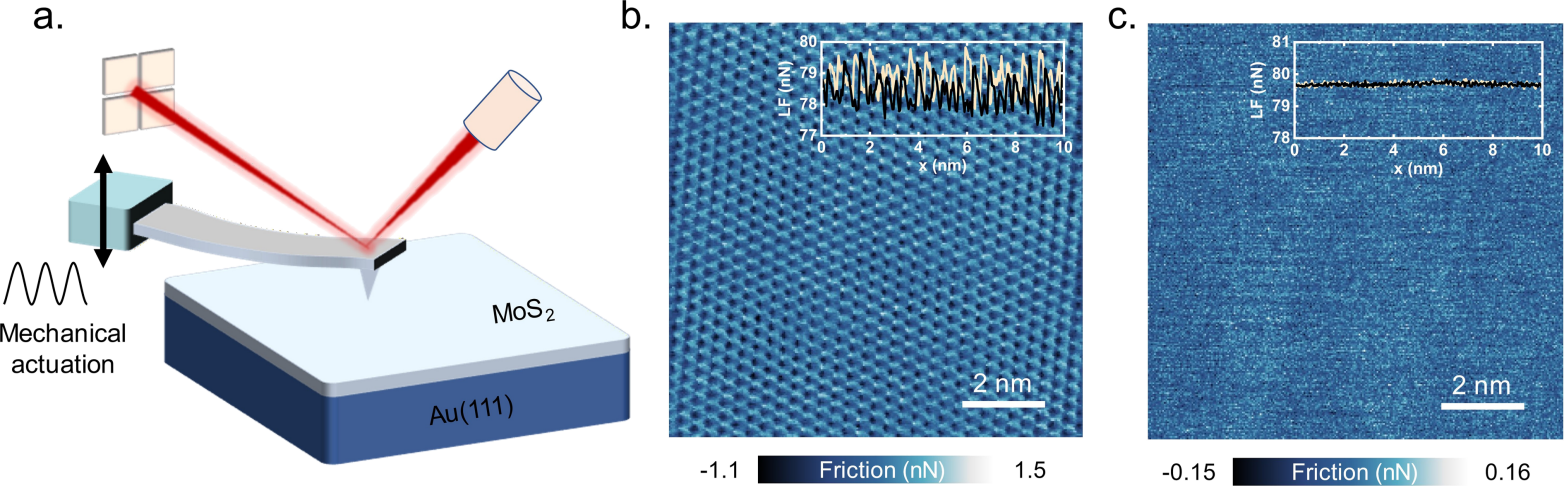
(a) A schematic diagram showing the experimental setup for friction measurements under external actuation on MoS_2_/Au(111) under ultrahigh vacuum. (b) Friction force map without actuation and (c) with actuation at 554 kHz. Inset: friction loop extracted along the central line of the corresponding friction map.

The experimental setup for friction measurements is illustrated in Figure 1a. The experiments were performed at room temperature using a custom-built beam-deflection atomic force microscope (AFM) system under ultrahigh vacuum (UHV). A MoS_2_ flake on a Au(111) surface was scanned by an AFM tip under UHV conditions. Monolayer MoS_2_ flakes were synthesized following established protocols [15,26–27]. The mechanical actuation was implemented by applying a vertical sinusoidal excitation to the cantilever base through a piezoelectric actuator, introducing out-of-plane vibrations to the tip–sample contact. The friction force is calculated as half the difference between the forward and backward lateral force signals. In the absence of external actuation (Figure 1b), the friction force map exhibits a periodic stick–slip pattern, with a periodicity of approximately 0.315 nm, which corresponds to the lattice constant of MoS_2_. Although the lattice mismatch between MoS_2_ and Au(111) implies the existence of a moiré superstructure, such long-range features are not clearly resolved in the lateral force signals. This is attributed to the strong interfacial coupling, which suppresses moiré-induced corrugations and leaves the atomic-scale periodicity as the dominant contribution to friction [26].

When actuation at an specific frequency of 554 kHz is applied, the stick–slip instability is effectively suppressed, as shown in Figure 1c. The mean friction force drops from 0.2 nN to near-zero level (0.006 nN). This transition is further evidenced by the individual friction loops shown in the insets. While the lateral force fluctuations are prominent in the unexcited state, they decrease to the noise level when the vibration is active. Quantitatively, the root-mean-square of the lateral force is reduced from 0.5 to 0.06 nN, confirming the successful suppression of atomic-scale stick–slip. These results indicate that the external vibration provides sufficient energy for the tip to overcome the local potential barriers. As a result, the tip slides in a nearly continuous manner rather than by stick–slip jumps, marking the onset of a dynamic structural superlubricity state.

**Figure 2:**
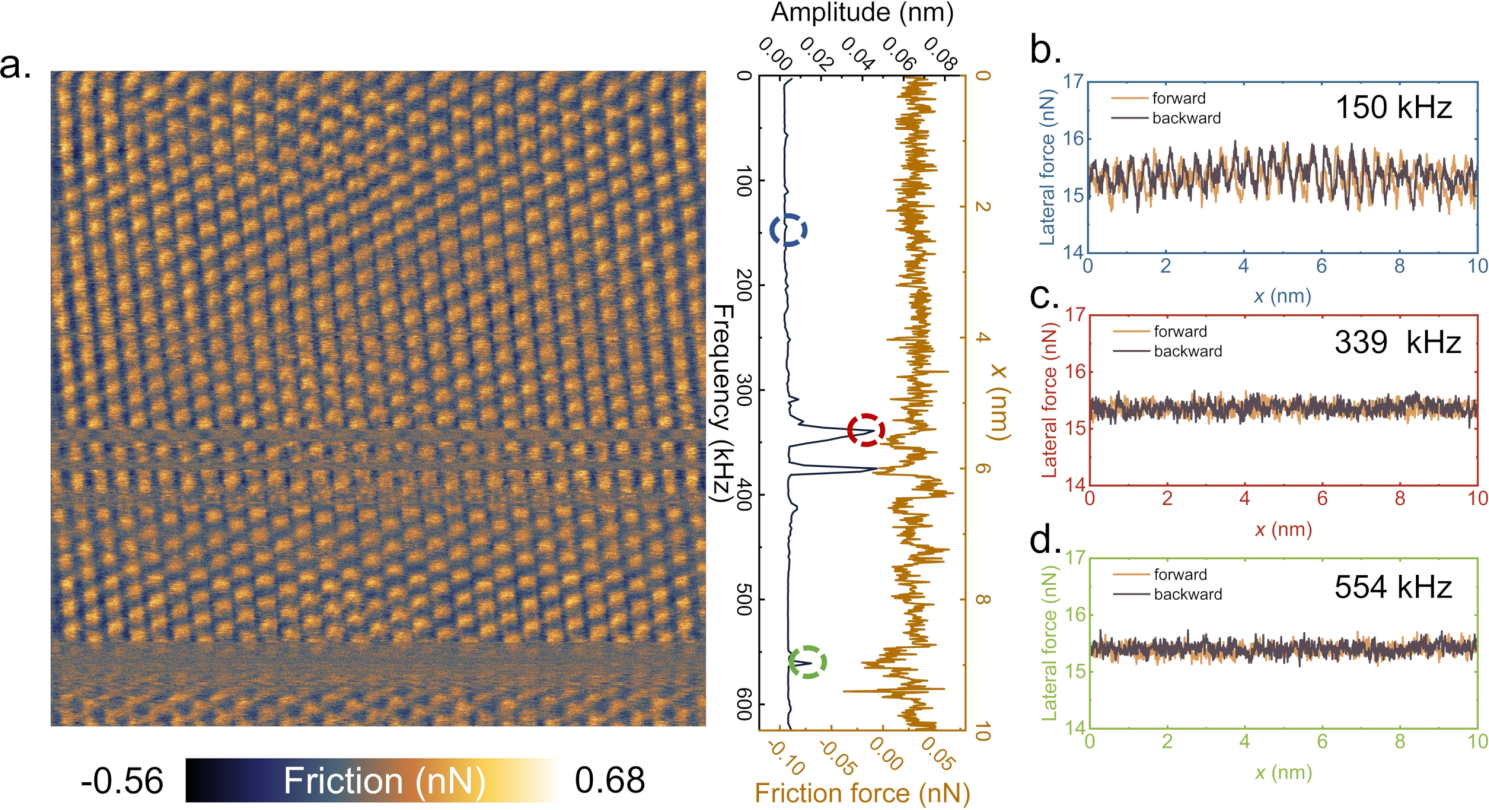
Effect of mechanical actuation on friction. (a) Friction force image measured as a function of actuation frequency in the range of 0 to 625 kHz. Scan parameters: 1024 × 1024 pixels, 0.2 nm excitation amplitude, and 2.6 nN normal load. (b–d) Forward (yellow) and backward (black) lateral force traces at actuation frequencies of 150, 339, and 554 kHz.

To systematically investigate the frequency dependence of the dynamic structural superlubricity, a frequency sweep from 0 to 625 kHz was performed with 256 sampling points (Figure 2a). The recorded tip vibration amplitude during frequency sweep and the mean friction line profiles are displayed on the right panel of the map. The results suggest a clear correlation between significant friction drops and the specific frequencies where the tip vibration amplitude increases. This indicates that the friction reduction is driven by a resonance-like coupling between the tip–sample contact and the external actuation.

To examine the atomic-scale friction in detail, individual friction loops were extracted from the frequency sweep at three representative frequencies (Figure 2b–d), also marked by circles in Figure 2a. At 150 kHz (Figure 2b), which is far from any resonance peak, the lateral force trace exhibits a distinct stick–slip pattern with significant hysteresis. In contrast, at 339 and 554 kHz (Figure 2c,d), where the vibration amplitude is high, the stick–slip instability is effectively suppressed. At these frequencies, the forward and backward lateral force traces almost overlap, and the friction is reduced to the noise floor. This frequency-selective behavior suggests that the suppression of stick–slip is a resonance-driven process, involving the mechanical eigenmodes of the sliding contact.

**Figure 3:**
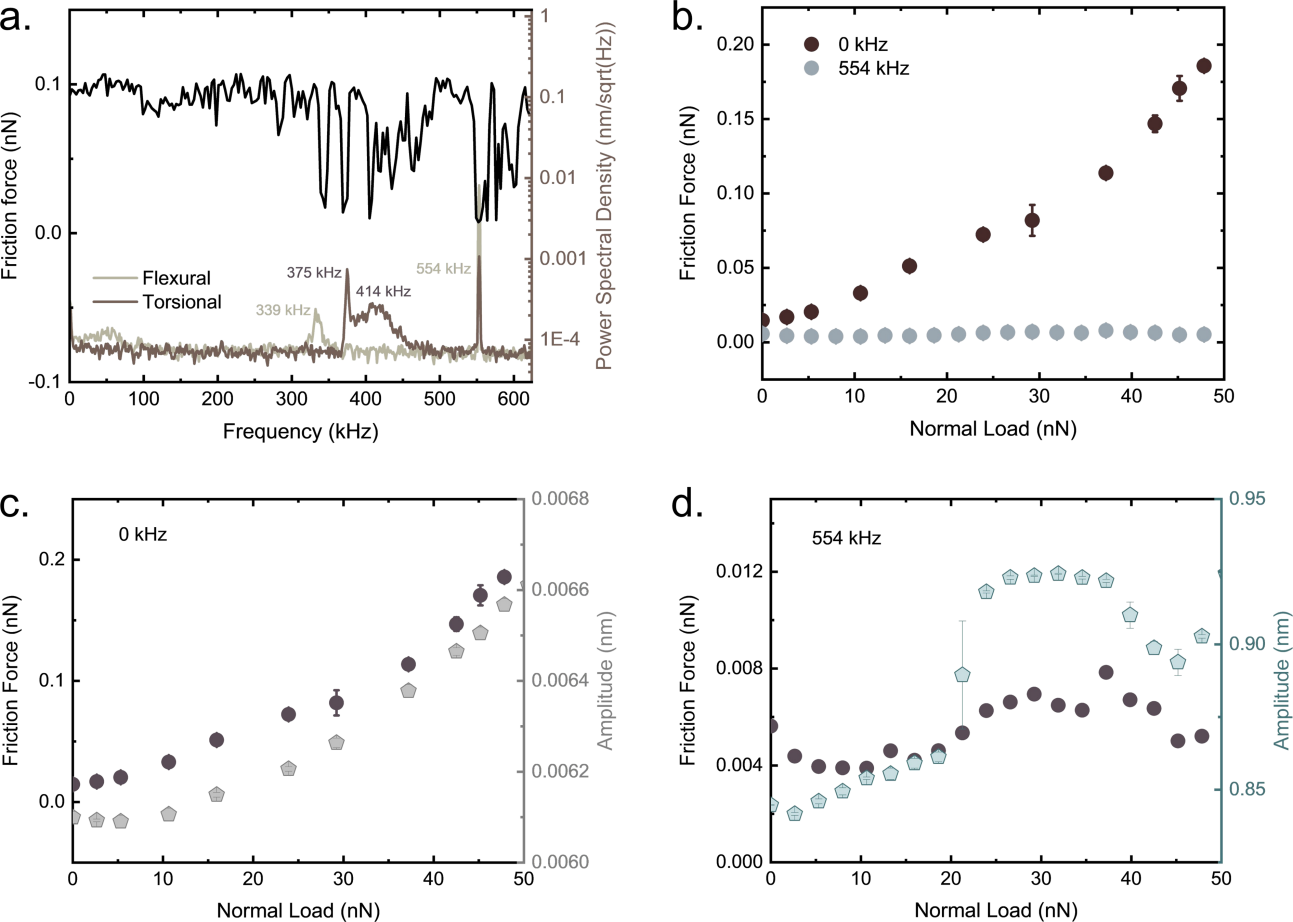
(a) Friction measured as a function of actuation frequency for a normal load of 2.6 nN. Right axis: corresponding thermal noise spectra of the vertical (gray) and torsional (brown) oscillations of the cantilever sliding on the MoS_2_/Au(111) surface at a frequency of 554 kHz. (b) Normal load dependence of friction at different actuation frequencies. (c) Mean friction (left axis) and tip amplitude (right axis) as functions of the applied normal load for actuation frequencies of 0 kHz and (d) 554 kHz.

To clarify the physical origin of the observed friction reduction, the frequency-dependent friction was compared with the thermal noise spectra of the cantilever. Figure 3a shows the mean friction force (black) alongside the flexural (gray) and torsional (brown) thermal noise power spectral densities. For comparison, the thermal noise spectra of the free cantilever are provided in Figure S1 of Supporting Information File 1.

Through modal identification, the friction minima at 339 and 554 kHz are assigned to the second- and third-order flexural contact resonance frequencies (*f*_c2_ and *f*_c3_, respectively), while the peak at 414 kHz corresponds to the first-order torsional contact resonance (*f*_t1_). Due to experimental constraints, the first-order flexural resonance was not detected because of a high-pass filter used for signals below 100 kHz. Notably, a distinct feature observed near 375 kHz remains not fully understood. We propose that this peak may arise from potential couplings related to the moiré superstructure; however, its origin remains to be further investigated.

The stability of this superlubric state was investigated over a wide range of normal loads. Figure 3b compares the load dependence of friction at 0 and 554 kHz. In the absence of external vibration (0 kHz), the friction force increases linearly with the applied normal load, following the characteristic Amontons–Coulomb behavior for atomic-scale contacts. In contrast, at 554 kHz, the friction remains suppressed near the noise floor throughout the investigated load range, confirming that the dynamic structural superlubric state remains stable even under high normal loads. In addition, the scanned area was characterized both before and after the friction measurements. No noticeable changes in the MoS_2_ surface were observed in the lateral force images after the experiments, indicating that the monolayer MoS_2_ maintained its structural integrity throughout the measurements. These observations demonstrate the excellent durability of the MoS_2_ flakes under the experimental conditions.

To further understand the relationship between friction and tip vibration, the load was analyzed together with the vibration amplitude. At 0 kHz, the friction rises steadily with the load, while the vibration amplitude remains at the noise level, as shown on right axis of Figure 3c. The minor increase in amplitude at high loads is attributed to the increased vertical deflection of the cantilever. At 554 kHz, however, the mean friction remains near-zero regardless of the load. The corresponding tip vibration amplitude remains consistently high (right axis of Figure 3d), confirming that sustained mechanical excitation effectively assists the tip in overcoming interfacial potential barriers. This prevented the tip from being trapped in potential wells even under high normal loads, maintaining the superlubric state.

## Discussion

**Figure 4:**
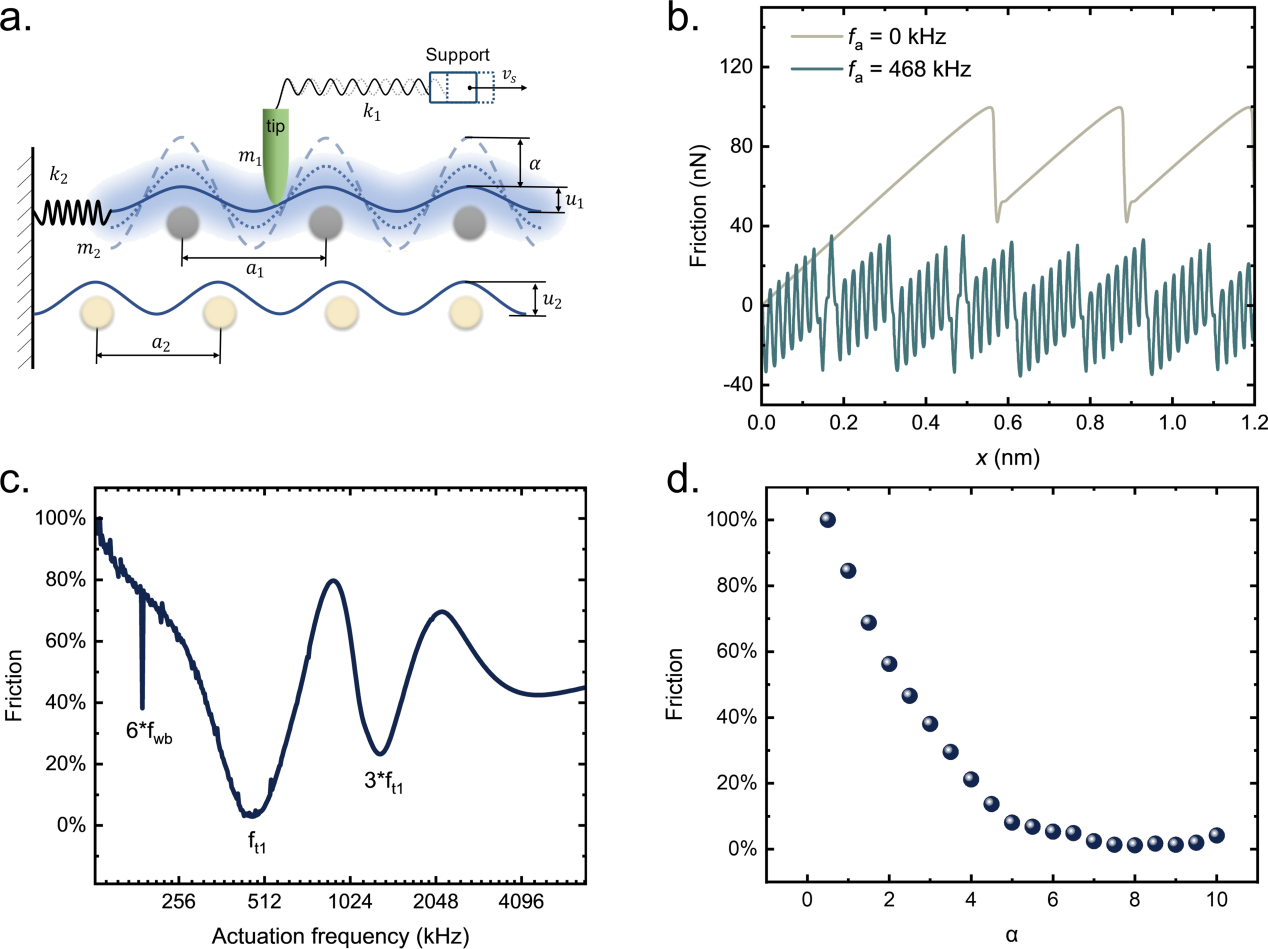
(a) A schematic diagram showing a one-dimensional PF model with a tip sliding on a composite structure comprising two periodic layers of MoS_2_ and Au(111). The top MoS_2_ layer exhibits perturbed energy corrugation stemming from out-of-plane external actuation. (b) A comparison of stick–slip friction with and without vibration. Mean friction as a function of (c) actuation frequency and (d) amplitude of perturbation.

Numerical simulations based on a modified one-dimensional phononic friction (PF) model were performed to elucidate the dynamic structural superlubricity observed on the MoS_2_/Au(111) surface, as shown in Figure 4a [28–30]. In the model, the top potential *V*_1_ with a MoS_2_ lattice constant of *a*_1_ and an amplitude of *u*_1_ describes the tip–MoS_2_ interaction. This is superimposed by a bottom potential *V*_2_ representing the MoS_2_–Au(111) interaction, characterized by a substrate periodicity of *a*_2_ and an amplitude of *u*_2_. The stiffness *k*_t_ denotes the lateral spring constant of the cantilever, and *k*_2_ represents the local interfacial stiffness between the MoS_2_ and the gold substrate. As the cantilever support moves at a constant velocity of *v*_s_, the tip and the MoS_2_ flake are displaced by *x*_1_ and *x*_2_, respectively, representing the tip displacement and the in-plane deformation of the top layer. *m*_1_ and *m*_2_ are the effective masses of the tip and the moiré, respectively.

To account for the mechanical actuation, the potential energy landscape of the MoS_2_ layer undergoes a time-dependent perturbation with a amplitude of α and an actuation frequency of *f*_a_ [31–32]. Therefore, the potential energy combined with vibration 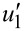 is:


[1] $${u^{\prime }}_{1}=u_{1}\left[1+\alpha \sin\left(2\pi f_{\text{a}}t\right)\right].$$

Thus, the total potential *V* of the dynamic friction system, including the tip–MoS_2_ and MoS_2_–Au(111) interactions (*V*_1_ and *V*_2_), the spring potential energy of the cantilever, and the moiré, is given by:


[2] $$\begin{array}{c} V={u^{\prime }}_{1}\cos\left[\frac{2\pi \left(x_{1}-x_{2}\right)}{a_{1}}\right]+u_{2}\cos\left[2\pi \left(\frac{x_{1}}{a_{2}}-\frac{x_{1}}{a_{1}}+\frac{x_{2}}{a_{1}}\right)\right]\\ +\frac{1}{2}k_{1}{\left(v_{\text{s}}t-x_{1}\right)}^{2}+\frac{1}{2}k_{2}x_{2}^{2}. \end{array}$$

The dynamics of the system can be described by the Langevin equation of motion (see Section S2 in Supporting Information File 1 for more details),


[3]

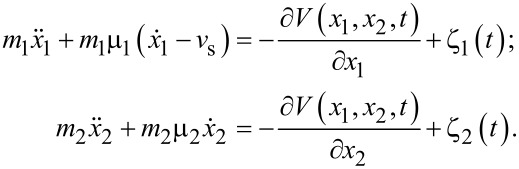



The friction force is given by *F*_f_ = *k*_1_(*v*_s_*t* − *x*_1_), and the resulting system of equations is integrated numerically using the fourth-order Runge–Kutta method. The simulation results for the instantaneous friction with and without excitation are compared in Figure 4b. To ensure consistency with the experimental findings, the potential amplitudes were set to *u*_1_ = 30 eV and *u*_2_ = 0.06 eV. The relatively small value of *u*_2_ reflects the minimal contribution of the moiré superstructure to the overall friction, resulting in the moiré-scale modulation not being clearly resolved in the calculated friction traces. Without excitation (0 kHz), the system exhibits a periodic stick–slip pattern with a periodicity of 0.315 nm, corresponding to the atomic lattice of MoS_2_. Upon applying a 468 kHz excitation, the friction force drops from an initial 66.63 nN to near-zero levels. In this state, the instantaneous friction force signal represents a superposition of the washboard frequency [28–29,33] and the external actuation frequency, as resolved by fast Fourier transform analysis (see Section S4 and Figure S3 in Supporting Information File 1). The dependence of the mean friction on the actuation frequency was further quantified through a frequency sweep (Figure 4c). The calculated friction exhibits several distinct drops. The first drop corresponds to the sixth harmonic of the washboard frequency [34], while the second and third drops align with the first-order (468 kHz) and third-order torsional contact resonances of the system, respectively (see Section S4 in Supporting Information File 1 for the determination of torsional contact resonance frequency in the PF model). As this is a one-dimensional model, the calculation specifically captures the torsional dynamics of the tip–sample contact, while vertical resonances are not included. These findings confirm that mechanical actuation at contact resonance modes effectively triggers a transition to a dynamic structural superlubric state, in good agreement with the experimental observations in Figure 2 and Figure 3. Furthermore, increasing the perturbation amplitude α leads to a rapid decay in the mean friction, which approaches zero and eventually saturates, as shown in Figure 4d. A larger vibration amplitude is equivalent to a lowering in the effective normal load or barrier height. The external energy provided by the vibration promotes tip escape from the atomic potential wells, effectively suppressing the stick–slip instability and leading to the robust friction reduction as observed experimentally.

## Conclusion

In summary, we have demonstrated active and reversible friction control at the MoS_2_/Au(111) interface using external mechanical actuation under ultrahigh vacuum. Our experimental results reveal that the transition from stick–slip to dynamic structural superlubricity is dictated by the precise coupling between the excitation frequency and the cantilever’s mechanical modes. Remarkably, this resonance-induced superlubric state exhibits stability across a wide range of normal loads, effectively overcoming the traditional load-dependent increase in friction typically observed at the atomic scale. By using mechanical vibration rather than electrical or thermal methods, this approach avoids the inherent limitations of Joule heating, material degradation, or the requirement for interfacial conductivity discussed earlier.

Numerical simulations using a modified phononic friction model consistently support our findings, confirming that the modulation of the interfacial potential at torsional contact resonance frequency assists the tip in overcoming the corrugation, thereby suppressing stick–slip instabilities. Although the dominant resonance modes are clearly assigned, the specific friction drop at 375 kHz warrants further clarification. Investigating the coupling between high-order vibrations and the moiré superstructure may resolve this unexplained feature. This work bridges the gap between macroscopic mechanical control and 2D interface engineering, offering a practical pathway for enhancing the performance and operational lifetime of next-generation NEMS and atomically precise technologies.

## Supporting Information

The Supporting Information includes the thermal noise spectra of the free cantilever, a detailed list of phononic friction model simulation parameters, and the methodology for determining torsional contact resonance frequency.

File 1 Experimental and calculation details.

## Data Availability

Data generated and analyzed during this study is openly available in Zenodo at https://doi.org/10.5281/zenodo.19552234.
