# Supplementary material for: Vibration-induced dynamic structural superlubricity on MoS2/Au(111) under ultrahigh vacuum
Source: Beilstein J Nanotechnol. 2026 Aug 6;17:1039–46. doi: 10.3762/bjnano.17.71 (PMC13454882; doi:10.3762/bjnano.17.71)
Supplement: Supplementary file 1 [file Beilstein_J_Nanotechnol-17-1039-s001.pdf]

BEILSTEIN JOURNAL OF NANOTECHNOLOGY

Supporting Information

for

Vibration-induced dynamic structural superlubricity on MoS_2/Au(111) under ultrahigh vacuum

Shuyu Huang, Yiming Song, Antoine Hinaut, Gema Navarro-Marín, Antonio Cammarata, Ernst Meyer and Thilo Glatzel

Beilstein J. Nanotechnol. **2026,** *17,* 1039–1046. doi:10.3762/bjnano.17.71

Experimental and calculation details

License and Terms: This is a supporting information file under the terms of the Creative Commons Attribution License (https://creativecommons.org/licenses/by/4.0). Please note that the reuse, redistribution and reproduction in particular requires that the author(s) and source are credited and that individual graphics may be subject to special legal provisions.
The license is subject to the *Beilstein Journal of Nanotechnology* terms and conditions: (https://www.beilstein-journals.org/bjnano/terms)

S1. Thermal noise spectra of the free cantilever

The thermal noise spectra of cantilever were recorded in the free state (far from the sample surface) under ultrahigh vacuum. Figure S1 presents the power spectral density of the flexural and torsional oscillations.

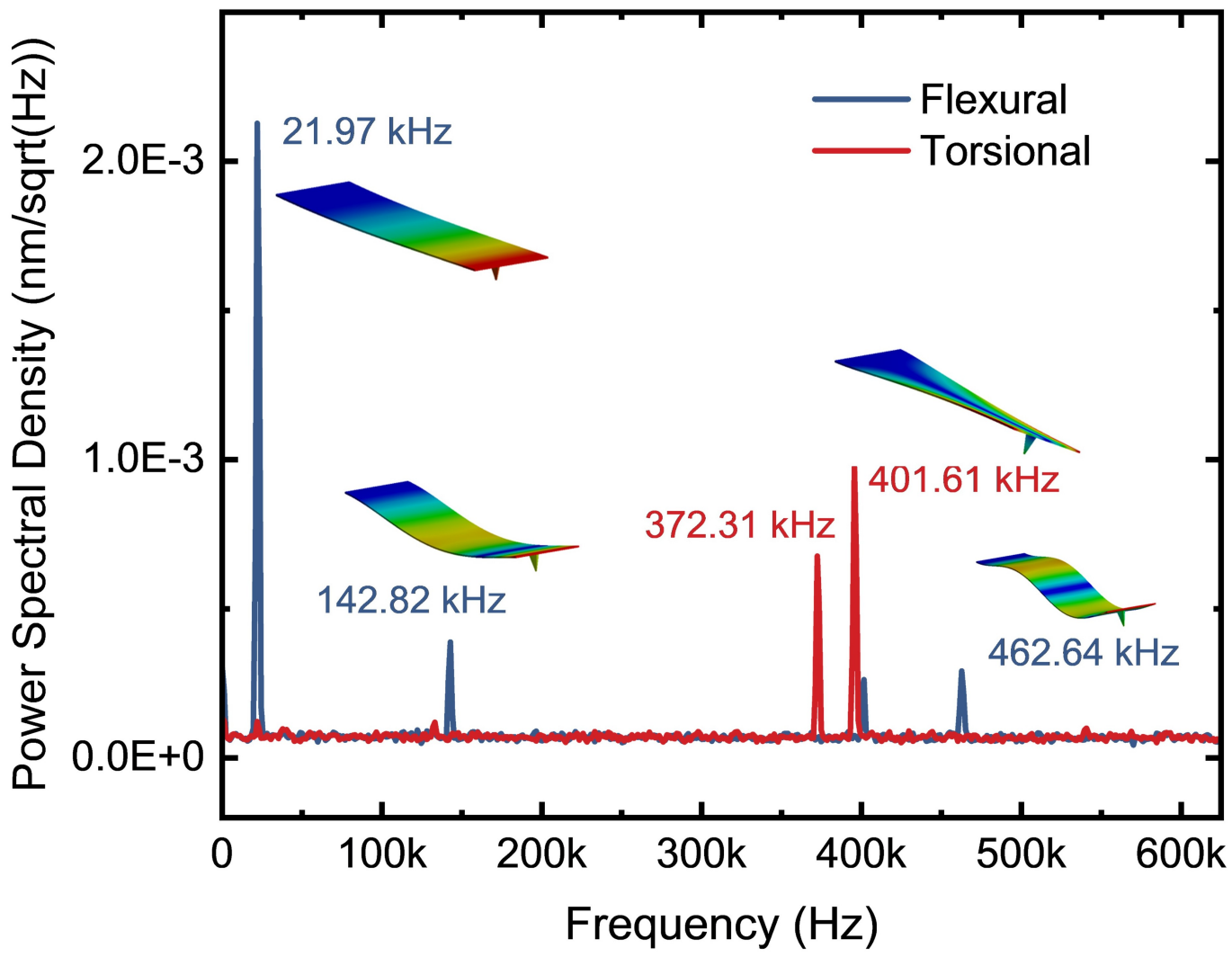

Figure S1: Thermal noise power spectral density flexural (blue) and torsional (red) vibration modes. Resonance peaks corresponding to the first three flexural modes and the first torsional mode are labeled.

The flexural resonance frequencies are identified at 21.97, 142.82, and 462.64 kHz, corresponding to the first-, second-, and third-order bending modes, respectively. The first-order torsional mode is observed at 401.31 kHz. Additionally, as mentioned in the main text, a distinct but unidentified peak appears at 372.31 kHz. The origin of this specific feature is not yet fully understood and may from internal cantilever resonances; it remains a subject for further investigation. These free-resonance values serve as the reference for identifying the contact resonance shifts described in the main text.

S2. Details and parameters of the phononic friction model

The dynamics of the tip–MoS_2-Au(111) system are governed by the Langevin equations of motion presented in the main text. In these equations, μ_1 and μ_2 are the damping coefficients representing the energy dissipation during sliding. To ensure numerical stability while maintaining the physical characteristics of the oscillating system, we set the damping to be sub-critical with the damping

ratio of 0.6. Therefore where $\mu_i = 0.6 \times 2\sqrt{k_i/m_i}$. The thermal noise terms $\zeta_1(t)$ and $\zeta_2(t)$ are defined by the fluctuation–dissipation relation $\langle \zeta_i(t)\zeta_i(t')\rangle = 2m_i\mu_i k_B T\delta(t-t')$. For simplification, the temperature T was set to 0 K in the present simulations. Numerical integration was performed using the fourth-order Runge–Kutta algorithm with a spatial resolution of 2000 sampling points per MoS_2 lattice constant (a_1). Structural and mechanical parameters, including the lattice constants of MoS_2 and Au(111) as well as the effective cantilever stiffness and mass, were determined directly from experimental measurements. The complete set of parameters used in the phononic friction (PF) model is summarized in Table S1.

Table S1: Parameters in the PF model calculations.

Parameter	Symbol	Value	Unit
MoS_2 lattice constant	a_1	3.15	Å
Au(111) lattice constant	a_2	2.88	Å
cantilever stiffness	k_1	419.73	N/m
moiré stiffness	k_2	419.73	N/m
effective mass (tip)	m_1	6.59×10^{-11}	kg
Effective mass (moiré)	m_2	6.59×10^{-12}	kg
tip–MoS_2 potential amplitude	u_1	30	eV
MoS_2-Au(111) potential amplitude	u_2	0.06	eV
perturbation amplitude	α	5	-
actuation frequency	f_a	468	kHz
sliding velocity	v_s	10	µm/s
damping coefficients 1	μ_1	3.03×10^{-3}	1/ns
damping coefficients 2	μ_1	9.58×10^{-3}	1/ns
simulation temperature	T	0	K

S3. Fast Fourier transform analysis of calculated instantaneous friction

To resolve the dynamic components of Figure 4b in main text, a fast Fourier transform (FFT) was performed on the calculated instantaneous friction traces.

As shown in Figure S2, the frequency spectrum distinguishes two primary components: the washboard frequency ($f_{wb} = v_s/a_1$), arising from the tip traversing the atomic lattice potential, and the external actuation frequency (f_a = 468 kHz). The distinct resolution of these peaks confirms that the

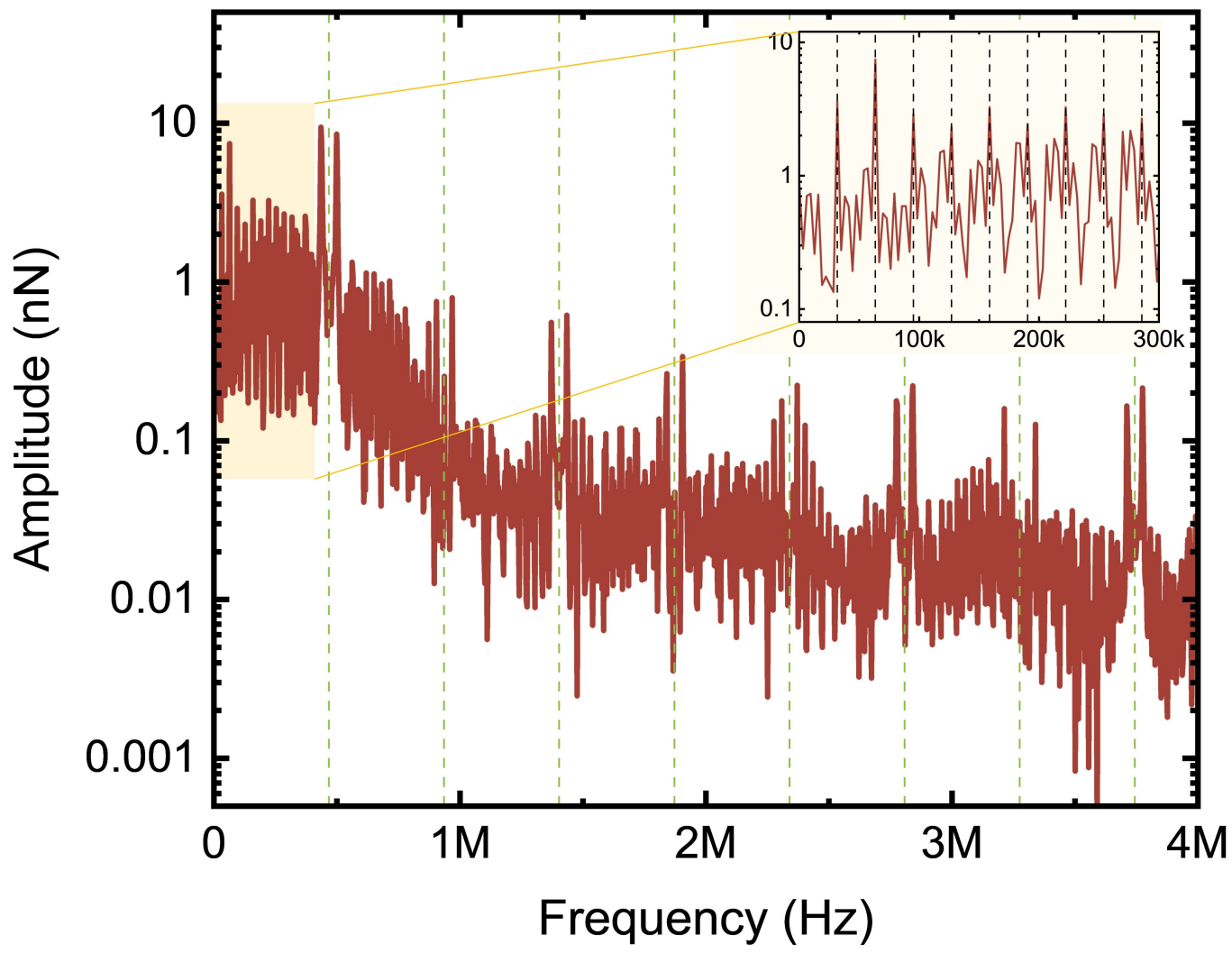

Figure S2: The FFT spectra of the instantaneous friction under 468 kHz actuation. Green dashed lines denote f_a and its harmonics. Inset: Magnified 0–300 kHz range, where black dashed lines identify the washboard frequency (f_{wb} = 31.75 kHz) and its harmonics.

friction reduction results from the dynamic superposition of mechanical modulation and potential corrugation.

S4. Determination of torsional contact resonance frequency in calculation

The torsional resonance frequency of the cantilever in its free state was set to 410.61 kHz in calculation, consistent with the experimental measurement. Upon establishing contact with the MoS_2 surface, the total system stiffness increases due to the additional interfacial contact stiffness (k_u), shifting the resonance to a higher frequency (f_c):

$$k_u = \frac{\partial^2 V(x_1, x_2, t)}{\partial x_1^2}. \tag{S1}$$

In the presence of external actuation, k_u is not a constant value but fluctuates periodically as the tip oscillates within the modulated potential landscape. The instantaneous contact stiffness is defined by the second derivative of the interaction potential:

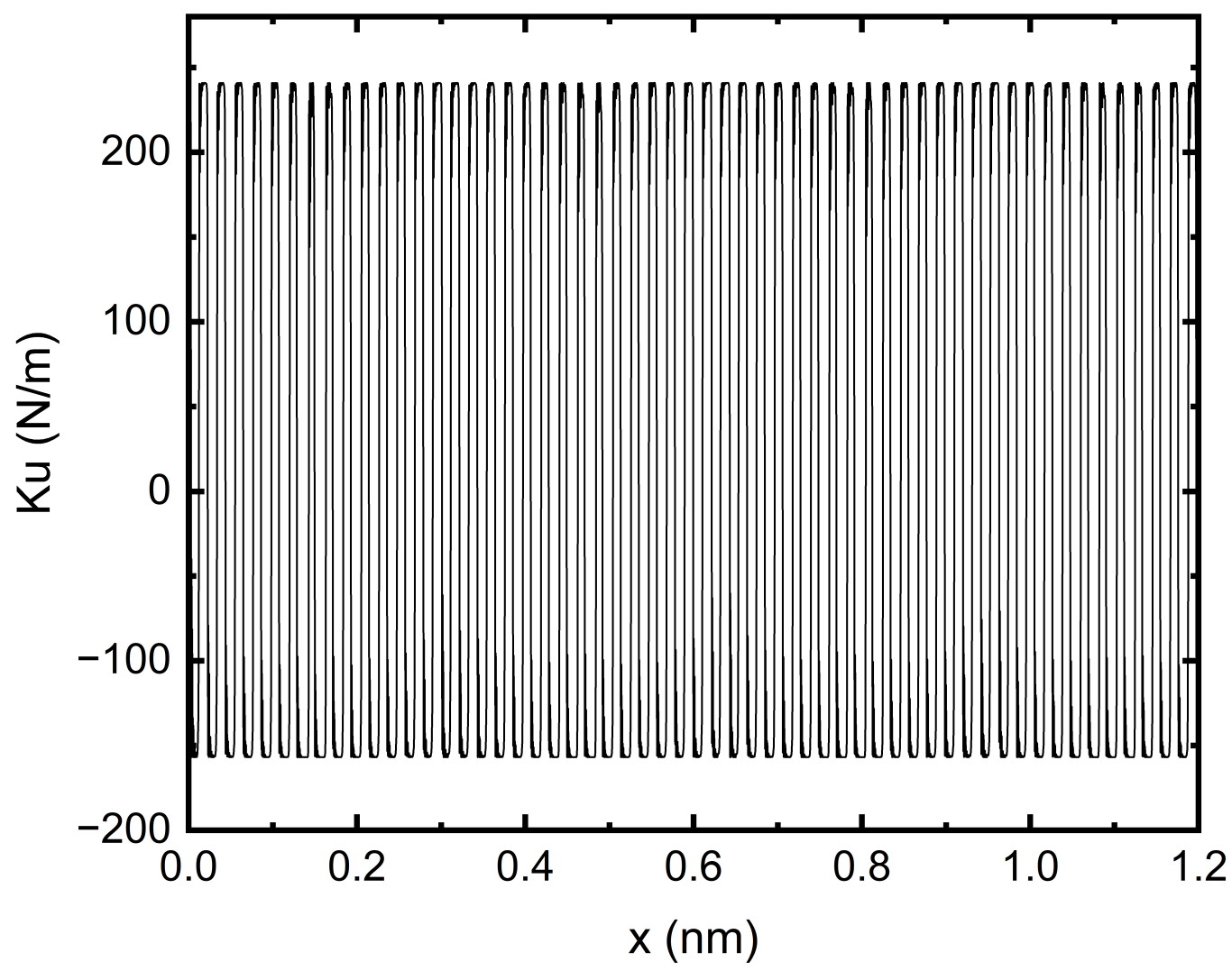

Figure S3: Calculated instantaneous contact stiffness k_u as a function of time under 468 kHz vibrational actuation.

The contact resonance frequency for the PF model was estimated by evaluating the time-averaged contact stiffness. As shown in Figure S3, for an actuation frequency of 468 kHz, the mean value of k_u is approximately 149.57 N/m. The resulting torsional contact resonance frequency f_c can then be estimated using the effective spring–mass relation:

$$f_c = \frac{1}{2\pi}\sqrt{\frac{k_1 + k_u}{m_1}} \tag{S2}$$

Substituting the simulation parameters ($k_1 = 419.73$ N/m, $m_1 = 6.59 \times 10^{-11}$ kg, and $\bar{k}_u \approx 149.57$ N/m) yields a contact resonance frequency of approximately 467.71 kHz. This value closely aligns with the applied 468 kHz excitation, confirming that the significant friction reduction observed in the simulation results from the resonance suppression of stick–slip instabilities.
